# Short-Term Monocular Deprivation Enhances Physiological Pupillary Oscillations

**DOI:** 10.1155/2017/6724631

**Published:** 2017-01-09

**Authors:** Paola Binda, Claudia Lunghi

**Affiliations:** ^1^Department of Translational Research on New Technologies in Medicine and Surgery, University of Pisa, Pisa, Italy; ^2^CNR Neuroscience Institute, Pisa, Italy

## Abstract

Short-term monocular deprivation alters visual perception in adult humans, increasing the dominance of the deprived eye, for example, as measured with binocular rivalry. This form of plasticity may depend upon the inhibition/excitation balance in the visual cortex. Recent work suggests that cortical excitability is reliably tracked by dilations and constrictions of the pupils of the eyes. Here, we ask whether monocular deprivation produces a systematic change of pupil behavior, as measured at rest, that is independent of the change of visual perception. During periods of minimal sensory stimulation (in the dark) and task requirements (minimizing body and gaze movements), slow pupil oscillations, “hippus,” spontaneously appear. We find that hippus amplitude increases after monocular deprivation, with larger hippus changes in participants showing larger ocular dominance changes (measured by binocular rivalry). This tight correlation suggests that a single latent variable explains both the change of ocular dominance and hippus. We speculate that the neurotransmitter norepinephrine may be implicated in this phenomenon, given its important role in both plasticity and pupil control. On the practical side, our results indicate that measuring the pupil hippus (a simple and short procedure) provides a sensitive index of the change of ocular dominance induced by short-term monocular deprivation, hence a proxy for plasticity.

## 1. Introduction

Recent studies have shown that activity in early visual cortex can be altered by a short period of monocular deprivation (MD) in adult humans. Specifically, after a few hours of monocular deprivation, ocular dominance unexpectedly shifts in favor of the deprived eye [1–7]. For example, monocular deprivation has dramatic perceptual consequences on the dynamics of binocular rivalry (a particular form of bistable perception that engages strong competition between the monocular signals [8]): following deprivation, the deprived eye dominates rivalrous perception for twice as long as the nondeprived eye, indicating a strong shift of ocular dominance in favor of the deprived eye [2, 3]. This apparently counterintuitive effect reflects a compensatory reaction of the visual system to the transient impoverishment of monocular visual input that is likely mediated by an upregulation of contrast-gain control mechanisms of the deprived eye (this hypothesis is supported by evidence that short-term monocular deprivation increases apparent contrast of the deprived eye [2]). These results indicate that the adult human visual cortex retains a high degree of homeostatic plasticity that takes place in the early levels of visual processing, since short-term monocular deprivation modulates the earliest component of the Visual Evoked Potential [1].

Evidence from animal studies has suggested that a key determinant for adult visual cortical plasticity is the balance between intracortical excitation and inhibition [9]. For instance, the critical period for ocular dominance plasticity is regulated by the maturation of specific GABAergic circuits [10, 11], suggesting that the decreased plastic potential of the visual cortex observed in adulthood can be determined by an increase of inhibition [12]. Consistent with this hypothesis, ocular dominance plasticity can be reinstated in adult animals by decreasing GABAergic inhibition (pharmacologically [13] or through environmental manipulations [14–16]). A recent study has shown that following 2.5 hours of monocular deprivation GABA concentration (measured by means of Magnetic Resonance Spectroscopy at 7-Tesla) drops in the adult primary visual cortex and across subjects the decrease in GABA levels highly correlates with the boost of deprived eye during binocular rivalry [4]. In agreement with the animal literature, these results strongly suggest that a release of GABAergic inhibition plays a crucial role in mediating homeostatic plasticity in adult humans.

Ocular dominance plasticity in adult animals can also be reinstated by increasing excitation. Specifically, three classes of excitatory neurotransmitters have been found to enhance visual cortical plasticity: serotonin [17], acetylcholine (Ach) [18], and norepinephrine (NE) [19, 20]. However, there is no evidence at present about a role of excitatory signaling modulating adult visual plasticity in humans.

The animal literature has recently highlighted how cortical excitability may be monitored through a simple, noninvasive, and yet sensitive measure: the diameter of the pupil [21]—resonating with a long tradition of studies in human participants [22].

It is well known that a range of stimuli can evoke pupil constrictions and dilations [23]: not only light increments and decrements, but also equiluminant stimuli [evoking a transient constriction [24]] and visual or nonvisual stimuli capable of evoking an orienting response [accompanied by pupil dilation, [25]]. Pupil dilations also accompany task effort, both physical work [e.g., [26]] and mental effort [27]. However, when stimulation is kept to a constant and minimal level and no task is assigned, the pupil still shows variations in size. These take the form of quasiperiodic slow oscillations, sometimes termed hippus [23, 28–30].

In the mouse, these alternations of pupil constriction and dilation effectively track the responsiveness of the cortex to sensory stimuli [21, 26, 31, 32]. Specifically, dilations are coupled with desynchronized activity across neural populations and increased sensitivity to visual/somatosensory stimulation, both time-locked to the change of activity in different classes of inhibitory interneurons [26] and to signaling in the norepinephrine and acetylcholine systems [33].

In primates, low-frequency oscillations of pupil size have been studied in diverse contexts and often linked to arousal levels, although the interpretation of such link and its relevance to cortical excitability are not straightforward. There is a large body of work associating very slow and very large pupil size changes with diminished arousal or sleepiness in humans [28, 30], yet pupil dilations are generally associated with increased arousal [22, 27, 34, 35] and prompt orienting to sensory stimuli [25]. Moreover, slow pupil waves can accompany epileptic seizures characterized by abnormally increased cortical excitability [36, 37]. In general, recent work has convincingly shown that the relationship between arousal levels and pupil size is well explained by a coupling of pupil diameter with activity in the Locus Coeruleus, the subcortical nucleus responsible for NE release [38, 39].

The importance of NE for adult cortical plasticity on the one hand and the tight relationship between NE tone and pupil diameter on the other inspired us to ask whether adult cortical plasticity is accompanied by a systematic change in the dynamics of pupil diameter.

As done in past experiments on adult MD, we assess the plasticity effect by means of binocular rivalry, comparing eye dominance before and after eye-patching. We choose not to measure pupil dynamics during binocular rivalry, but in a separate session with no visual stimulation. This choice is motivated by prior work showing that pupil size is sensitive to the dynamics of binocular rivalry [40, 41] and that pupil responses to visual stimuli may be larger/smaller when the stimulus representation in the visual cortex is enhanced/suppressed, for example, enhanced during focused attention [42–46] or suppressed during saccadic eye movements [47, 48]. Thus, it is expected that pupil behavior during binocular rivalry changes after MD, simply as a result of its modifying the rivalrous interplay between the eyes [2] and affecting cortical responses to the deprived eye [1]. We avoid this confound by measuring pupil dynamics at rest: in the dark, with participants staring straight-ahead while no visual or otherwise sensory stimulus is manipulated.

## 2. Methods

### 2.1. Subjects

10 subjects (5 females, mean age ± standard deviation: 24.57 ± 2.06) participated in the study. All subjects were naïve to the experiment, had normal or corrected-to-normal visual acuity, and did not show strong eye dominance (ratio between the two eyes binocular rivalry mean phases durations ≤ 1.5). Experimental procedures were approved by the regional ethics committee [Comitato Etico Pediatrico Regionale—Azienda Ospedaliero-Universitaria Meyer—Firenze (FI)] and are in line with the Declaration of Helsinki; participants gave their written informed consent.

### 2.2. General Procedure

We measured binocular rivalry and pupil diameter before and after 2 hours of monocular deprivation. The measurements obtained before the deprivation were used as baseline (two 180 sec experimental blocks for binocular rivalry, one 120 sec block of pupillary measurement).

**Figure 1:**
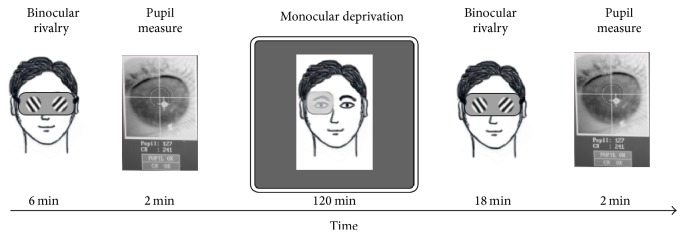
Diagram of the experimental procedure. Baseline binocular rivalry dynamics and pupillary measurements were obtained before deprivation (2 × 180 sec binocular rivalry blocks, 2 minutes of pupil measurement in total darkness). Short-term monocular deprivation was achieved by having observers wear a translucent eye-patch over the dominant eye for 2 hours. Immediately after eye-patch removal, 4 × 180 sec binocular rivalry blocks were acquired within a temporal interval of 18 minutes. Binocular rivalry tests were followed by 2 minutes of pupillary measurement in the dark.

During the two hours of monocular deprivation, observers watched a movie while sitting in front of a TV screen at a distance of 80 cm. Immediately after eye-patch removal, we measured binocular rivalry for 18 minutes in four separate 180 sec blocks separated by a two-minute break to allow the subject to rest: this is the standard protocol used in the previous studies on MD from our laboratory [2, 3, 49]. Two minutes after the last binocular rivalry block (20 minutes after eye-patch removal), we measured the pupillary diameter in one 120 sec block. A diagram of the experimental procedure is shown in Figure 1. Binocular rivalry and pupil size were measured in different setups, both housed in dark and quiet experimental rooms. This protocol allowed for collecting a single measure of pupil size before and (20 minutes) after MD; future work is necessary to measure the changes of pupil behavior immediately after eye-patch removal (when the effect on binocular rivalry is maximum) and later on (comparing the decay of the MD effect on pupil behavior versus binocular rivalry).

### 2.3. Monocular Deprivation

Previous reports [2, 3] have shown that monocular deprivation induces stronger shifts in eye-dominance when the dominant eye is patched compared to the nondominant eye. For this reason, in the current study monocular deprivation was performed by patching the dominant eye for 2 hours. Eye-dominance was assessed using binocular rivalry: the dominant eye was defined as the eye showing the longer mean phase duration in the baseline (predeprivation) measurements. The eye-patch was made of a translucent plastic material that allowed light to reach the retina (attenuation 15%) but completely prevented pattern vision, as assessed by the Fourier transform of a natural world image seen through the eye-patch.

### 2.4. Apparatus and Procedure: Binocular Rivalry

Visual stimuli were generated by the VSG 2/5 stimulus generator (CRS, Cambridge Research Systems), housed in a PC (Dell) controlled by Matlab (The Mathworks) scripts. Visual stimuli were two Gabor Patches (Gaussian-vignetted sinusoidal gratings), oriented either 45° clockwise or counterclockwise (size: 2*σ* = 2°, spatial frequency: 2 cycles/degree of visual angle, and contrast: 50%), presented on a uniform background (luminance: 37.4 cd/m2, C.I.E.: 0.442 0.537) in central vision with a central black fixation point and a common squared frame to facilitate dichoptic fusion. Visual stimuli were displayed on a 20-inch Clinton Monoray (Richardson Electronics Ltd., LaFox, IL) monochrome monitor, driven at a resolution of 1024 × 600 pixels, with a refresh rate of 120 Hz. In order to achieve dichoptic stimulation, observers viewed the display at a distance of 57 cm through CRS ferromagnetic shutter goggles that occluded alternately one of the two eyes each frame.

Observers sat in front of the monitor wearing the shuttering goggles. After an acoustic signal (beep), the binocular rivalry stimuli appeared. Subjects reported their perception (clockwise, counterclockwise, or mixed) by continuously pressing with the right hand one of three keys (left, right, and down arrows) of the computer keyboard. Another acoustic signal (three beeps) signaled the end of each 180 sec experimental block. At each experimental block, the orientation associated to each eye was randomly varied so that neither subject nor experimenter knew which stimulus was associated with which eye until the end of the session, when it was verified visually.

### 2.5. Apparatus and Procedure: Pupillometry

An EyeLink 1000 system (SR Research, Canada) monitored two-dimensional eye position and pupil diameter with an infrared camera mounted below a monitor screen (Barco Calibrator, 40 × 30 cm), which was only used for calibrating the eye tracker (13-point calibration routine). The eye-monitor (and eye-camera) distance was maintained to 57 cm by means of a chin rest. Eye-tracking data were acquired at 1000 Hz and streamed from the EyeLink to a Mac Pro 4.1 through the EyeLink toolbox for Matlab [50]. The setup is hosted in an experimental room illuminated only by the monitor screen. Pupil diameter values were output online by the EyeLink system (computed with internal algorithms) and we only used Matlab to receive and store them together with gaze position estimates.

Recording sessions lasted 120 seconds, during which participants faced the monitor screen set to minimum luminance (<1 cd/m2) with no fixation point or other prominent features they might focus on. They were instructed to relax accommodation and stare straight-ahead, trying to avoid eye, head, and body movements and to keep blinking to a minimum.

Gaze position and pupil diameter were always recorded from the nondeprived eye (although both eyes were unpatched at the time of recording, that is, viewing was always binocular during eye-tracking). Note that pupillary responses are consensual, the two pupils reacting simultaneously and by the same amount in all but pathological cases. Therefore, we do not expect any change of the present results if the deprived eye pupil was recorded instead. Pupil diameter measures were transformed from pixels to millimeters using an artificial 4 mm pupil, positioned at the approximate location of the subjects' left eye.

### 2.6. Analyses: Binocular Rivalry

The perceptual reports recorded through the computer keyboard were analyzed with custom Matlab scripts. During binocular rivalry, visual perception oscillates between the monocular images and periods of exclusive dominance of one of the two rivalrous stimuli are sometimes interleaved with periods in which the observer perceives a mixture of the two images, called mixed percepts. In order to quantify ocular dominance, for each subject and each experimental block, we computed the average duration of exclusive dominance of each stimulus, called mean phase duration, as well as the average duration of mixed percepts. The 180 sec blocks acquired after monocular deprivation were binned as follows: 0–8 min and 10–18 min. In order to obtain an index of the effect of deprivation, we computed the Deprivation Index (DI) described in [4], which summarizes the change in eye-dominance (defined as the ratio between the deprived and nondeprived eye mean phase durations) induced by monocular deprivation (see (1)). DI = 1 indicates no change in ocular dominance compared to predeprivation measurements, DI < 1 indicates increased dominance of the deprived eye, and DI > 1 indicates increased dominance of the nondeprived eye.

(1) $$\begin{array}{c} \text{Deprivation Index}=\frac{DepEye_{pre}}{DepEye_{post}}*\frac{NonDepEye_{post}}{NonDepEye_{pre}}. \end{array}$$

The deprivation indexes obtained for each of the two experimental blocks measured after eye-patch removal were compared against the value of 1 using a one-sample, two-tailed *t*-test. Mean phase durations of each eye obtained before and after deprivation were compared using a two-tailed paired-samples *t*-test. The Bonferroni correction for multiple comparisons was applied.

### 2.7. Analyses: Pupillometry

Eye-tracking data were analyzed with custom Matlab scripts. Pupillometry data consisted of 120 × 1000 time points (120 seconds at 1000 Hz). These included signal losses, eye-blinks, and other artifacts, which we eliminated before assessing the oscillatory behavior of the pupil. The majority of these artifacts were excluded based on pupil size being 0 (e.g., during eye-blinks). However, this left time points with highly instable pupil size measures (e.g., disturbances from eye-lashes) as well as short intervals, typically preceding and following a blink, where the pupils acquired very small or very large values. We cleaned these out by means of custom software that identifies and excludes the changes of pupil size that are too fast to be physiologically meaningful. Specifically, the algorithm starts by identifying time points where the rate of change of pupil diameter (pupil difference in the unit of time) is larger than a threshold (set to the 90th percentile of pupil change rate of each participant). These time points are labeled as artifacts and temporarily replaced with the average pupil diameter; then the procedure is repeated ten times. The first round will exclude any time point where the pupil recording is unstable as well as the first time point where pupil size suddenly drops (a blink) or increases (disturbance from eye-lashes). Iterating the procedure allowed for further eliminating the short intervals where the pupil happens to stabilize at an artefactual value (which typically last few ms).

**Figure 2:**
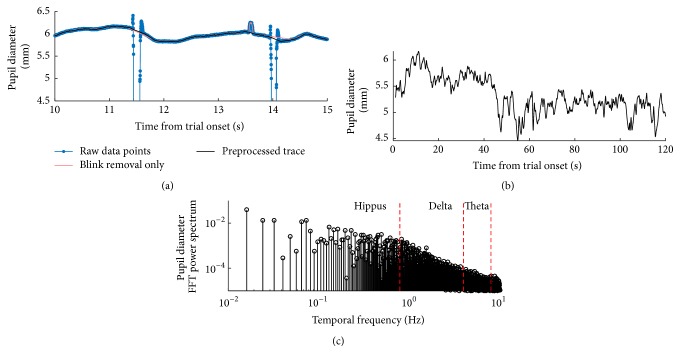
Preprocessing of pupillometry data. (a) Segment of pupil recording showing three artifacts: two typical blink artifacts and a short dilation artifact (eye-lashes disturbance). All three were removed by our preprocessing algorithm (black line). For comparison, the result of applying a more standard blink removal algorithm is shown by the red line. (b) Full preprocessed trace, same block from which the segment in (a) was extracted. (c) FFT spectrum of the pupil recording in (c), highlighting the frequency bands used in the main analysis. For this trace, the PUI value was 3.07.

This custom procedure proved to be more effective than a standard blink removal algorithm, which eliminates 500 ms worth of data every time the pupil drops below 2 mm (see example in Figure 2(a)). We verified that the number of detected artifacts was indistinguishable before and after deprivation (paired *t*-test on the percentage of excluded data samples, *t*(9) = 1.54,  *p* = 0.1586) and that the main results could be reproduced using either of the two algorithms (see caption of Figure 4).

We then used linear interpolation to replace data points labeled as artifacts and we proceeded to extract the low-frequency components of pupil oscillations by means of fast Fourier transform (applied after subtracting the mean pupil size). For each 120 sec trace we computed the energy in three contiguous frequency bands: hippus (0–0.8 Hz), delta (0.8–4 Hz), and theta (4–8 Hz). As an alternative quantification of the energy in the hippus range, we also computed the Pupillary Unrest Index or PUI [29]: the sum of absolute changes in pupil diameter based on a sample frequency of 1.5625 Hz (exactly the same definition used in [29]).

Horizontal and vertical gaze position data from time points where an artifact in pupil diameter was detected (see above) were excluded. Deviations from screen center were computed and the sign of horizontal gaze shifts was flipped for subjects where the right eye was recorded. In this way, a positive horizontal gaze shift implies a shift in the nasal direction and a negative shift implies a shift in the temporal direction. We took the average across time of horizontal and vertical gaze shifts as a measure of systematic gaze deviations and we estimated fixation instability by means of Bivariate Contour Ellipse analysis. This amounts to defining an ellipse around the *x*, *y* coordinates of gaze position samples. Its area is defined by

(2) $$\begin{array}{c} BCEA=2k\sigma_{H}\sigma_{V}{\left(\;1-R^{2}\;\right)}^{0.5}, \end{array}$$

where the constant *k* relates to the percentage of data points that fall within the ellipse. As in previous studies, for example, [51], we set *k* = 1.14 so that 68.2% of the data points fall within the ellipse.

Using a series of paired *t*-tests we compared the average pupil diameter, the power in the hippus/delta/theta range, the PUI, the horizontal and vertical gaze shifts, and the fixation instability values obtained after deprivation versus before deprivation. We took the difference between the two values as an estimate of the deprivation effect, which we correlated with the effect of deprivation observed on binocular rivalry (quantified as the “Deprivation Index” defined in (1)).

## 3. Results

We measured the dynamics of binocular rivalry and the diameter of the pupil in a group of healthy adult volunteers before and after a short period (2 hours) of monocular deprivation during which observers wore a translucent eye-patch over the dominant eye.

### 3.1. Binocular Rivalry

**Figure 3:**
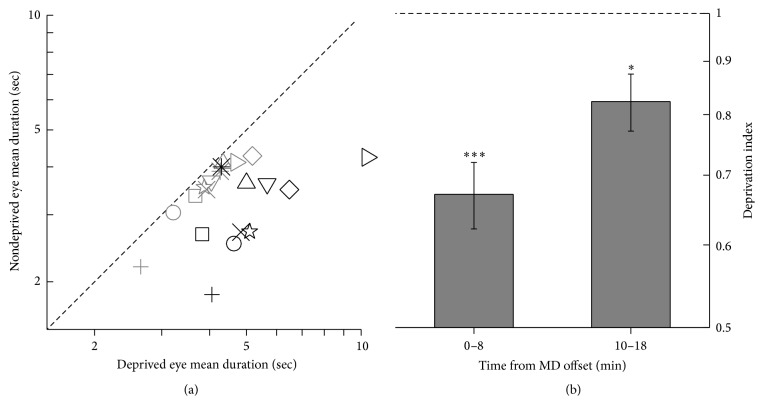
Binocular rivalry results. (a) Scatter plot of the individual subjects' mean phase durations for the deprived and nondeprived eye obtained before (light grey symbols) and during the first 8 minutes after monocular deprivation (black symbols). (b) Deprivation Index (see (1) in Methods) summarizing the increase in deprived eye-predominance for the first 8 minutes after eye-patch removal and for the interval between 10 and 18 minutes after deprivation. The Deprivation Index value of 1 (designated by the dashed line) would indicate no change in ocular dominance after deprivation; values smaller than one indicate increased deprived eye-predominance. Error bars represent 1 ± s.e.m.; asterisks represent statistical significance (^*∗∗∗*^*p* < 0.001,  ^*∗*^*p* < 0.05).

Mean phase durations of the deprived and nondeprived eye measured before eye-patching and during the first 8 minutes after short-term monocular deprivation are reported in Figure 3(a). Consistently with previous reports [2, 3], two hours of monocular deprivation boosted the deprived eye signal, resulting in increased deprived eye-predominance on eye-patch removal. After deprivation, mean phase durations of the deprived eye increased significantly (baseline mean phase duration (mean ± 1 s.e.m.) = 3.99 ± 0.23 s, mean phase duration after deprivation = 5.43 ± 0.6 s, two-tailed paired-samples *t*-test: *t*(9) = −2.87,  *α* = 0.025, Bonferroni corrected *p* = 0.036), while mean phase durations of the nondeprived eye decreased (baseline mean phase duration (mean ± 1 s.e.m.) = 3.57 ± 0.19 s, mean phase duration after deprivation = 3.14 ± 0.24 s, two-tailed paired-samples *t*-test: *t*(9) = 3.63,  *α* = 0.025, Bonferroni corrected *p* = 0.011) compared to predeprivation measurements.

A direct measure of the deprived eye increase in perceptual predominance induced by monocular deprivation is summarized by the* Deprivation Index *(see (1) in Methods), and it is shown in Figure 3(b). The Deprivation Index was significantly lower than 1 in both measurements obtained during the first 8 minutes after deprivation offset (mean ± 1 s.e.m. = 0.67 ± 0.05, one-sample, two-tailed *t*-test,* H*_0_: *X* = 1,  *t*(9) = −6.71,  *α* = 0.025, Bonferroni corrected *p* < 0.001), indicating that monocular deprivation robustly shifted eye dominance in favor of the deprived eye compared to predeprivation levels. The effect of deprivation decayed after eye-patch removal and was significant, albeit smaller, for measurements obtained in the interval from 10 to 18 minutes after eye-patch removal (mean ± 1 s.e.m. = 0.82 ± 0.05, one-sample, two-tailed *t*-test,* H*_0_: *X* = 1,  *t*(9) = −3.42,  *α* = 0.025, Bonferroni corrected *p* = 0.016).

### 3.2. Pupil

**Figure 4:**
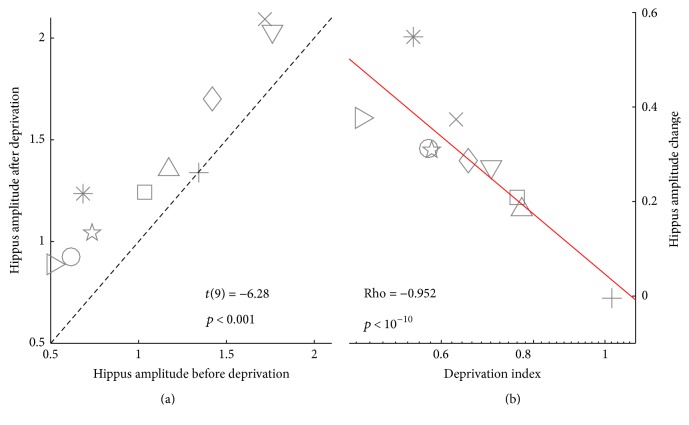
Pupillometry results and correlation with binocular rivalry. (a) Scatter plot of the FFT energy in the 0–0.8 Hz range, measuring the amplitude of the pupil hippus, before deprivation versus 20 minutes after deprivation. The text inset shows the result of a paired *t*-test comparing values on the *x*- and *y*-axis. Similar values are obtained using the alternative preprocessing algorithm (blink removal only: *t*-test comparing before/after hippus amplitude: *t*(9) = 6.743,  *p* < 0.001). (b) Difference of FFT energy in the hippus range across deprivation, plotted against the binocular rivalry Deprivation Index (see (1)). The text inset shows Spearman's rank correlation coefficient Rho between values on the *x*- and *y*-axis (note that Spearman's Rho is insensitive to whether the axes are logarithmic or linear). The thick red line is the best-fit linear function through the data points. Each symbol is one subject, consistent across panels (a)-(b) and with Figure 3(a).

Pupillary diameter was measured in the dark immediately before eye-patching and 20 minutes after eye-patch removal (after the binocular rivalry measurements). Pupillary oscillations in the low-frequency range (<0.8 Hz, known as the “hippus” range) are enhanced after monocular deprivation, compared to the baseline measure acquired before applying the eye-patch (Figure 4(a); paired *t*-test *t*(9) = 6.278,  *p* < 0.001). Similar values are obtained using an alternative preprocessing algorithm (blink removal only: *t*-test *t*(9) = 6.743,  *p* < 0.001).

This is also seen as an increase of the Pupillary Unrest Index, which provides an alternative measure of slow oscillations. The PUI goes from an average 1.10 ± 0.15 (mean and s.e.m. across subjects) before deprivation to 1.39 ± 0.14 after deprivation (paired *t*-test: *t*(9) = 4.545,  *p* < 0.01).

On the other hand, oscillations in the delta (0.8–4 Hz) and theta ranges (4–8 Hz) are unaffected by deprivation (delta before: 0.30 ± 0.03, after: 0.31 ± 0.03,  *t*(9) = 0.583,  *p* = 0.574; theta before: 0.07 ± 0.01, after: 0.07 ± 0.01,  *t*(9) = 0.151,  *p* = 0.883). The average pupil diameter shows a nonsignificant tendency towards decreasing after deprivation (average pupil diameter before: 6.02 ± 0.21 mm, after 5.88 ± 0.16 mm,  *t*(9) = −1.657,  *p* = 0.132).

### 3.3. Gaze Stability

While vertical gaze position was indistinguishable before/after deprivation, there was a tendency for horizontal gaze position to shift inward (nasally) after deprivation, from −0.59 ± 0.42 deg before to 0.27 ± 0.49 deg after; the effect is only significant before correcting for multiple comparisons (*t*(9) = 2.573,  *p* = 0.030). This marginally significant effect might be related to anomalies in the vergence eye movements occurring during the deprivation period, to be clarified by future studies.

More importantly, we find that the variability of gaze position (measured as the area of the Bivariate Contour Ellipse, BCEA) was not affected by deprivation, with similar BCEA values observed before and after MD (0.70 ± 0.24 deg^2^ and 0.41 ± 0.08 deg^2^, respectively, paired *t*-test *t*(9) = 1.500,  *p* = 0.168).

### 3.4. Correlation between Deprivation Effects on Pupillary and Binocular Rivalry Behavior

To test whether the effects of deprivation on binocular rivalry and slow pupil oscillations are related, we measured Spearman's correlation coefficient (Rho) between the binocular rivalry Deprivation Index (see (1)) and the increased power in the hippus range (Figure 3(b)). The two measures show a tight correlation (Spearman's Rho = −0.952,  *p* < 10^−10^, 95% Confidence Intervals, CI = from −0.805 to −0.988); the correlation remains high and significant using the alternative algorithm for preprocessing pupil (blink removal only: Spearman's Rho = −0.806,  *p* = 0.008, 95% CI = 0.359–0.952).

We also tested the correlation between the Deprivation Index and the other two indices of gaze behavior that showed at least a trend towards changing with deprivation: PUI, average pupil diameter, and shift of horizontal gaze (in the nasal direction). The correlation with PUI is weaker than with hippus power (Spearman's Rho = −0.418,  *p* = 0.232), indicating that FFT power gives a more precise quantification of the pupillary behavior. There is no correlation with either average pupil diameter (Spearman's Rho = 0.297,  *p* = 0.407) or horizontal gaze (Spearman's Rho = −0.2,  *p* = 0.584).

## 4. Discussion

By testing binocular rivalry before and after monocular deprivation, we find that MD transiently shifts ocular dominance in favor of the deprived eye, in line with previous work from our and other laboratories [1–7]. Upon completion of the binocular rivalry tests, we measured pupil size during two minutes of rest: with no visual stimulation, with participants sitting in the dark and performing no task except minimizing body and gaze movements. We find that the dynamics of the pupil are altered after MD, with increased amplitude of low-frequency oscillations, that is, enhanced hippus. This effect is specific for oscillations in the “hippus” range (slower than about 1 Hz, a time scale that is very similar to the frequency of perceptual oscillations during binocular rivalry), whereas faster oscillations (in the delta or theta ranges) are indistinguishable before/after MD, and so is the average pupil diameter.

Testing conditions also allowed us to check for the statistics of gaze position. This is important given that eye movements are known to influence both pupil dynamics [52] and binocular rivalry [53] and that increased frequency of eye movements could enhance pupil size oscillations. Our finding that the variability of gaze position is unaffected by MD speaks directly against this possibility.

The most important aspect of our results is the tight correlation between the effects of MD on our two very different measures, obtained minutes apart with different apparatus: pupillary hippus and increased eye-dominance of the deprived eye during binocular rivalry. We interpret this by suggesting that the change of visual cortical excitability induced by monocular deprivation [1, 4, 5] results in behavioral changes both during visual stimulation (as measured by binocular rivalry) and during rest (as indexed by pupillary oscillations). The tight correlation between psychophysics and pupillometry agrees with a growing body of literature showing that changes of cortical excitability can be accurately tracked by the variations of pupil size over time [21, 22] and our findings specifically agree with the observation that the change of pupil size (i.e., the first derivative of pupil diameter over time) is a better predictor than the raw pupil diameter [26, 54]. One limitation of the current study is the relatively small sample size (*n* = 10); even though the correlation between the change in pupillary hippus and ocular dominance is strong (Rho = 0.95), further experimental work is needed to confirm this result in a larger sample of participants.

We speculate that the key to understanding this close relationship between pupillary hippus and plasticity lies within the complex neural circuits that regulate the balance between inhibition and excitation in the cortex, where the neuromodulator norepinephrine plays a key role. Hippus amplitude is thought to depend on the imbalance of noradrenergic (NE) and cholinergic (Ach) transmission [55], with pupil dilations correlating tightly with activity in the NE-releasing Locus Coeruleus [38, 39]; at the same time, animal studies have implicated NE transmission in ocular dominance plasticity in the visual cortex [19, 20], suggesting the possibility that a change of NE tone might be responsible for both of the effects we observe. The neural circuitry linking NE to cortical excitability and plasticity is still unclear but current work being performed in animal models (especially mice) holds great potential for unraveling these complexities. For example, it has been recently shown that the activity of Vasoactive Intestinal Peptide-Expressing (VIP+) GABAergic interneurons and Somatostatin-Expressing (SOM+) interneurons in the primary visual cortex of mice is modulated during slow spontaneous pupillary oscillations [26]. Specifically, VIP+ interneurons are more active during pupil dilation than during constriction, while SOM+ interneurons show the opposite behavior. Interestingly, the VIP+/SOM+ circuit is also implicated in activating visual cortical plasticity in adult animals [16]. Moreover, ocular dominance plasticity is enhanced by physical exercise in both mice and humans [49, 56], and the effect in mice is linked to a selective modulation of VIP+ and SOM+ interneurons [56]. Taken together, these data indicate that there is partial overlap between neural circuits that are important for the regulation of ocular dominance plasticity and the regulation of slow pupil oscillations, and this overlap may help explain the correlation we observe between short-term plasticity and hippus. This is further supported by recent evidence that low-frequency pupil oscillations track changes in adrenergic and cholinergic activity in cortex [33]. Yet, the evidence to-date remains correlational, and any causal link might be sought for in future work.

The present work highlights how pupil behavior, a physiological parameter that can be continuously and noninvasively tracked with relatively simple apparatus, provides for a rich source of information. Not only does it provide objective and quantitative measures of responses to sensory stimuli [as we argued elsewhere, [46]], it also indicates the “internal state” of the individual [31]. Here we show that, by tracking pupil size during just two minutes while the participant is simply required to rest, one can obtain an index that is strongly correlated with ocular dominance plasticity, which, to be measured directly, requires substantial time and participants' collaboration. This could prove particularly important for probing visual cortical plasticity in clinical populations, where the patients' collaboration is difficult to obtain. One paradigmatic case would be the monitoring of neuroplasticity in young amblyopic children, as short-term homeostatic plasticity has been recently shown to be present in adult amblyopic patients [57] and to be predictive of the occlusion therapy outcome in anisometropic children [58].
