# Corrigendum to “Short-Term Monocular Deprivation Enhances Physiological Pupillary Oscillations”

**DOI:** 10.1155/2017/3634598

**Published:** 2017-09-24

**Authors:** Paola Binda, Claudia Lunghi

**Affiliations:** ^1^Department of Translational Research on New Technologies in Medicine and Surgery, University of Pisa, Pisa, Italy; ^2^CNR Neuroscience Institute, Pisa, Italy

In the article titled “Short-Term Monocular Deprivation Enhances Physiological Pupillary Oscillations” [1], an acknowledgement should be added as follows.

The research leading to these results was also funded by the Fondazione Roma under the Grants for Biomedical Research: Retinitis Pigmentosa (RP)-Call for proposals 2013 “Cortical Plasticity in Retinitis Pigmentosa: an Integrated Study from Animal Models to Humans”.
